# Validation of the French translation of the Dutch residency educational climate test

**DOI:** 10.1186/s12909-020-02249-4

**Published:** 2020-10-02

**Authors:** Mohamed Anass Majbar, Yassin Majbar, Amine Benkabbou, Laila Amrani, Abdeslam Bougtab, Raouf Mohsine, Amine Souadka

**Affiliations:** 1 Digestive Surgical Oncology Department, National Institute of Oncology, Ibn Sina University Hospital Centre, Rabat, Morocco; 2 Surgery Department, Faculty of Medicine. Mohammed V University in Rabat, Rabat, Morocco; 3 Faculty of Medicine, Sidi Mohamed Ben Abdellah University, Fes, Morocco

**Keywords:** Educational climate, Postgraduate medical education, Learning climate, Residency, Cross-cultural validation

## Abstract

**Background:**

The learning environment is one of the most influential factors in training of medical residents. The Dutch Residency Educational Climate Test (D-RECT) is one of the strongest instruments for measuring the learning environment. However, it has not been translated in French. The objective of this study is the psychometric validation of the DRECT French version.

**Material and methods:**

After translation of the D-RECT questionnaire into French, residents of five Moroccan hospitals were invited to complete the questionnaire between July and September 2018. Confirmatory factor analysis was used to evaluate the validity of the construct using the standardized root mean square residual (SRMR), the root mean square error approximation (RMSEA), the Comparative Fit Index (CFI) and the Tucker- Lewis Index (TLI). Reliability analysis was analysed using Internal consistency and Test-retest.

**Results:**

During the study period, 211 residents completed the questionnaire. Confirmatory factor analysis showed an adequate model fit with the following indicators: SRMR = 0.058 / RMSEA = 0.07 / CFI = 0.88 / TLI = 0.87. The French translation had a good internal consistency (Cronbach alpha score >  0.7 for all subscales) and a good temporal stability (correlation score between two measurements = 0.89).

**Conclusion:**

This French version has an acceptable validity of the construct, a good internal consistency and good temporal reliability, and may be used to evaluate the learning climate. Additional research is necessary in other French-speaking contexts, in order to confirm these results.

## Background

Postgraduate medical education takes place in most countries in the form of residency training. The objective of this training is to produce competent medical specialists capable of meeting population and country needs. The quality of resident training is therefore a major issue for decision-makers and educational leaders in medical schools and university hospitals.

Among influential elements in residency postgraduate medical training systems quality, the concept of environment, or climate of training becomes increasingly important in pedagogic research [1]. It includes many facets of resident training and reflects how individuals approach learning in clinical departments and incorporates their common perceptions on topics such as atmosphere, supervision, and learning. Learning environments are constructed through the interactions of learners with other health care workers and are influenced by organizational arrangements [1]. The measurement of learning climates can serve as a general indicator of the quality of a department’s education because of the versatility of its construction [2].

There are several instruments for evaluating the learning environment in the context of residency [3–8]. In a literature review, Soemantri et al. identified 31 different instruments dedicated to medical education, with only seven dedicated to residency [3]. The two most used instruments in this context are the “Dundee Ready Education Environment Measure” (DREEM) and the “Postgraduate Hospital Educational Environment Measure” (PHEEM) [2]. The DREEM was developed in Scotland to evaluate the learning environment in medical students, nursing students and residents [9]. Its strengths were its good internal consistency and its content validity [3]. The PHEEM was developed by Roff et al. to better assess the learning and teaching environments of doctors in training in the United Kingdom [10]. It includes 40 questions divided into three categories: the perception of autonomy, the perception of education and the perception of social support. PHEEM was considered suitable for use in the evaluation of postgraduate medical training because of its content validity, its high reliability and its ability to be used in different postgraduate contexts [3].However, these two instruments lack a clearly described theoretical basis, and their underlying factor structure is contested [2, 9, 11, 12]. For instance, the content and construct validities of DREEM were not established when applied in postgraduate settings [3] and the PHEEM has been described as having different subscales in different publications [11, 12].

Boor et al. considered that such a controversial subscale structure precludes any possibility of having a good assessment of the postgraduate environment [2]. To overcome the constraints, Boor and [2].al developed a new psychometric test based on qualitative research findings [2]. A final version called the Dutch Residency Educational Climate Test (DRECT) with 50 items was created. Then, Silkens et al. [13] showed that the learning climate could be assessed using 35 questions grouped into nine subscales. This new version of the DRECT was administered to 1537 residents in the Netherlands for validation. It showed good internal consistency and good fit for the factorial model.

Several studies have used the DRECT to assess the learning environment and its impact on resident training [14–18]. It was used successfully in quality improvement programs for residency systems [19]. It was also strongly correlated with the performance of teaching staff in clinical departments [20, 21] and with the occurrence of burn-out among residents [22].

In order to be accepted, the validation of the psychometric properties of a translated version must follow a rigorous process, especially when translating instruments from different countries and cultures. After the constitution of the research team, the original instrument is translated, usually using a forward-backward method and adapted to the new context. Then, the translated version is administered to a small group of the targeted population to ensure clarity of wording and meaning and instructions. The final version of the translated and adapted version is administered to a larger population to ensure its construct validity and reliability [23]. The psychometric properties of the revised DRECT have been validated only in the Philippines (English) and Columbia (Spanish) [16, 24] and have not been validated in French.

In Morocco, French is the second language after Arabic, and medical teaching is done only in French. Therefore, we undertook this study of psychometric validation of the French translation of DRECT.

## Material and methods

### Description of the DRECT

The modified DRECT includes 35 questions, divided into 9 sub-scales: “Educational atmosphere” (5 questions), “Teamwork” (3 questions), “Role of the speciality tutor” (6 questions), “Coaching and assessment” (6 questions), “Formal education” (4 questions), “Resident peer collaboration” (3 questions), “Work is adapted to residents’ competence” (3 questions), “Accessibility of supervisors” (3 questions) and “Patient sign-out” (2 questions). The question can be answered on a five point Likert-scale (1 = totally disagree, 2 = disagree, 3 = neutral, 4 = agree, 5 = totally agree) [13]. The total DRECT score is the mean of all 35 questions. The score of each subscale is the mean of the questions within the subscale.

### Translation and adaptation

The original questionnaire [13] was translated from English into French independently by two bilingual doctors (Forward translation). This translation was consolidated during a meeting of the research team. Then the French version was translated back to English by a professional translator (Backward translation). The translated questionnaires were evaluated during a meeting to reconcile the French and the English versions. The research team modified two questions to be more adapted with the local residency system (Supplementary material 1) [23, 25].

### Pre-test

The initial version was submitted to 10 residents [26], during face-to-face interviews with a member of the research team. For each question, residents were asked if the question was clear and understood, and to propose changes to improve the questionnaire [27]. All remarks were noted and evaluated by the research team and eventually incorporated to the final version of the questionnaire in French (Supplementary material 1).

### Distribution of the questionnaire

In addition to the French version of the DRECT, the final questionnaire included demographic and professional questions also in French. An electronic questionnaire was created using the Google Form platform (https://www.google.com/forms/about/) and was submitted to Moroccan residents between July 1 and September 30, 2018. In the absence of residents’ email databases in medical schools, it was not possible to directly target residents in a consistent manner. To overcome this difficulty, referent doctors were designated in each university hospital and they were responsible for distributing the form to residents. Participation in the study was voluntary and data was collected anonymously. The first page of the questionnaire contained detailed description of the study and its objectives and a question asking for the participants’ agreement to participate in the study. All participants gave their agreement via the google form.

### Statistical methods

Quantitative variables are expressed as mean and standard deviations, or medians and quartiles as appropriate. For DRECT answers, normality of data distribution was assessed by the asymmetry test and the Kurtosis test. Absolute asymmetry values less than 3 and Kurtosis values less than 10 are considered acceptable for confirmatory factor analysis [28]. Missing values ​​were replaced using the expectation-maximization (EM) technique. For the DRECT scores, the means and standard deviations of each item and of the nine subscales were calculated. In addition, for each item, discrimination (r_it_), or item-total-correlation for each of the 9 subscales were calculated. Item-total correlations above 0.4 were considered good [17].

### Evaluation of the construct validity

Confirmatory factor analysis (CFA) was used to evaluate the validity of the construct [26, 29, 30]. The fit of the model was evaluated by the following indices [29]: SRMR (standardized root mean square residual), RMSEA (root mean square error approximation), CFI (Comparative Fit Index) and TLI (Tucker- Lewis Index). Threshold values ​​for these indices were predetermined according to Brown’s recommendations [29, 30] (SRMR < 0.08 for a good fit and < 0.12 for an acceptable fit, RMSEA < 0.06 for a good fit and < 0.10 for an acceptable fit CFI and TLI >  0.95 for a good fit and >  0.90 for an acceptable fit).

Convergent validity was assessed using factor loadings (or regression coefficients) and average variance extracted (AVE). Factor loadings above 0.55 and AVE above 0.5 were considered satisfactory [29, 31]. Inter-scale correlation was used for discriminant validity. Correlations between subscales of 0.85 or above indicate poor discriminant validity [29].

### Reliability analysis

#### Internal consistency

Internal consistency was assessed using Cronbach Alpha test [32]. A result greater than 0.7 was considered satisfactory [33]. The internal consistency was measured for the entire DRECT questionnaire and for each of the nine subscales. In addition, Corrected item-total correlations were calculated to examine the homogeneity of each subscale. Corrected item-total correlation above 0.40 was considered satisfactory [34].

#### Test – retest

We invited 15 residents to respond to the questionnaire a second time to assess the stability of responses over time. A minimum of 2 weeks was necessary between the first and second measurements. Test-retest intraclass correlation coefficient greater than 0.6 was considered satisfactory [26].

IBM SPSS statistics 21 application was used for descriptive statistics, analysis of internal consistency, and test-retest reliability. The SPSS Amos Application Version 21 was used for confirmatory factor analysis.

## Results

During the study period, out of 695 residents contacted, 211 responded to the questionnaire (response rate of 30.3%). The characteristics of the participants are presented in Table 1. The mean age was 29.1 years (standard deviation 2.6). There were 97 men (46.0%) and 114 women (54.0%). First-year (25.6%), third year (24.6%) and fourth year (23.7%) residents were the most represented in the study. Finally, there were more residents in medical specialty (54.5%) than surgical (32.2%) or medical-surgical specialty (13.3%).

**Table 1:** Demographic characteristics of the population

Variables	Results
**Mean age** (Years, Standard Deviation)	29.11 (2.65)
**Gender**	
Men	97 (46.0%)
Women	114 (54.0%
**City**	
Fes	85 (40.3%)
Rabat	94 (44.5%)
Marrakech	8 (3.8%)
Casablanca	13 (6.2%)
Oujda	11 (5.2%)
**Year of residency**	
1	54 (25.6%)
2	34 (16.1%)
3	52 (24.6%)
4	50 (23.7%)
5	21 (10.0%)
**Type of speciality**	
Medical	115 (54.5%)
Surgical	68 (32.2%)
Medico-Surgical	28 (13.3%)

### DRECT scores

Of the 211 responses received, there was a form that contained two missing responses from the same resident, which represented less than 0.01% missing data. The mean score of the DRECT score was 3.21 (standard deviation 0.77). The mean scores for DRECT subscales ranged from 3 to 3.79. Table 2 shows the means and standard deviations for the 9 subscales of the DRECT. The mean score for DRECT items ranged from 2.66 to 3.94. All discrimination scores were above 0.4. Detailed descriptive statistics for each item of the DRECT is available in supplementary material 2.

**Table 2:** Results of the DRECT sub-scales with Cronbach alpha scores for all other studies that validated the revised DRECT

			Cronbach			
Subscale (number of items)	Mean (SD)	AVE	This study	Silkens et al. [13]	Pacifico et al. [16]	Dominguez et al. [24]
**Educational atmosphere** (5)	3.16 (0.92)	0.48	0.82	0.83	0.85	0.86
**Teamwork** (3)	3.07 (0.99)	0.58	0.79	0.79	0.93	0.84
**Role of specialty tutor** (6)	3.21 (1.02)	0.65	0.91	0.86	0.96	0.88
**Coaching and assessment** (6)	3.00 (1.05)	0.66	0.91	0.82	0.92	0.88
**Formal education** (4)	3.10 (1.04)	0.67	0.89	0.79	0.88	0.84
**Resident peer collaboration** (4)	3.54 (0.90)	0.61	0.81	0.84	0.92	0.84
**Work is adapted to residents’ competence** (3)	3.19 (0.90)	0.5	0.70	0.71	0.88	0.78
**Accessibility of supervisors** (3)	3.79 (0.95)	0.68	0.82	0.71	0.93	0.86
**Patient sign-out** (2)	3.04 (1.14)	0.79	0.88	0.78	0.91	0.88

*SD* standard deviation / Cronbach: Cronbach alpha results (> 0.7 is adequate) / *AVE*: average variance extracted (> 0.5 is adequate)

The minimum and maximum scores for each subscale are 1 and 5 respectively

### Confirmatory factor analysis

Table 3 shows the results of confirmatory factor analysis. Based on Brown’s recommendations, two indicators, SRMR (0.058) and RMSEA (0.07) had satisfactory results and two indicators, CFI (0.88) and TLI (0.87) had a result slightly below the recommended threshold. Table 3 also shows comparison of CFA results with all other studies that validated the revisited DRECT [13, 16, 24].

**Table 3:** Indicators of construct validity of confirmatory factor analysis of this study and all other studies that validated the revised DRECT

Indice	This study	Silkens et al. [13]	Pacifico et al. [16]	Dominguez et al. [24]
SRMR	0.058	0.04	–	0.06
RMSEA	0.07	0.04	0.108	0.06
CFI	0.88	0.92	0.84	0.84
TLI	0.87	0.91	0.82	0.82

*SRMR* Standardized Root Mean Square Residual (< 0.08 for a good fit); *RMSEA* root mean square error of approximation (< 0.06 for a good fit and < 0.10 for an acceptable fit); *CFI* Comparative Fit Index (> 0.95 for a good fit and > 0.90 for an acceptable fit); *TLI* Tucker- Lewis Index (> 0.95 for a good fit and > 0.90 for an acceptable fit)

For convergent validity, factor loadings ranged between 0.48 and 0.93 (supplementary material 2). Only one item had a factor loading below 0.55. All subscales had an AVE score above 0.5 (Table 2), with the exception of the subscale “Educational atmosphere” (0.48). For discriminant validity, Inter-scale correlations showed correlation factors of less than 0.85, indicating adequate discriminant validity (Table 4).

**Table 4:** Inter-scale correlations

	1	2	3	4	5	6	7	8	9
1. Educational atmosphere	1	0.6	0.65	0.64	0.66	0.33	0.65	0.62	0.44
2. Teamwork	–	1	0.6	0.69	0.58	0.33	0.72	0.46	0.49
3. Role of specialty tutor	–	–	1	0.79	0.84	0.23	0.77	0.59	0.7
4. Coaching and assessment	–	–	–	1	0.77	0.27	0.77	0.62	0.68
5. Formal education	–	–	–	–	1	0.28	0.74	0.57	0.74
6. Resident peer collaboration	–	–	–	–	–	1	0.38	0.32	0.23
7. Work is adapted to residents’ competence	–	–	–	–	–	–	1	0.61	0.7
8. Accessibility of supervisors	–	–	–	–	–	–	–	1	0.54
9. Patient sign-out	–	–	–	–	–	–	–	–	1

### Reliability analysis

Table 2 shows the Cronbach alpha test results for each subscale. All subscales showed a score greater than 0.7 (minimum 0.7, maximum 0.91). Corrected item-total correlation ranged from 0.355 to 0.813 (Table 2). Only one item in subscale “Work is adapted to residents’ competence “had a corrected item-total correlation below 0.4.

Of the fifteen residents contacted, thirteen answered the questionnaire twice. The correlation coefficient obtained was 0.89, indicating a good correlation between the two measurements.

## Discussion

Despite the importance of the learning environment concept, its use in the French-speaking countries is limited, probably due to the lack of a validated French version. We have therefore tried to address this issue through the validation of the French version of the Dutch Residential Educational Climate Test (DRECT). Statistical analysis showed that this translated version had good internal consistency, good temporal reliability and good construct validity with adequate convergent and discriminant validity.

The reliability of a psychological test concerns the accuracy of the instrument no matter what it measures [26]. Recent research has highlighted the use of two types of indices: internal consistency indices and test-retest stability indices. When the test is measured on a rating scale (like a Likert-style scale), the Cronbach alpha analysis is recommended [26]. The value of the alpha coefficient can vary between 0 and 1. The higher the scores, the more the instrument is judged to have a high level of internal consistency. On the other hand, a score too high (more than 0.95 for example) would indicate the presence of a redundancy in the elements which infers that some of them measure an overly narrow aspect of the concerned dimension. Values ​​between 0.70 and 0.85 are therefore generally preferred [26]. As for the other studies [13, 16, 24], the scores obtained varied between 0.7 and 0.91, indicating a good internal consistency of the French version. Test-retest indices were evaluated by asking subjects to fill the instrument twice. We asked 15 residents to complete the questionnaire twice, but only 13 responded. The correlation coefficient was 0.89, indicating good temporal stability. Test-retest has not been evaluated in previous DRECT creation or validation research.

Confirmatory factor analysis (CFA) is the recommended method for validating the factorial structure of a questionnaire [26, 29, 30]. When developing a new instrument in the social sciences, the factor structure is determined by the exploratory factor analysis, then the created instrument is validated by the confirmatory factor analysis. In the case of validation of a translation of an instrument, the confirmatory factor analysis is used directly, taking as a model the proposed structure of the original instrument. Although the CFA is strongly recommended, it is noteworthy that it is not systematically used for the validation of an instrument. Pinnock et al. used only internal consistency to validate the adaptation of the Boor’s DRECT in the Australian context [14]. Similarly, Caron et al. [35] only used internal consistency (Cronbach) to validate the French translation of PHEEM (Postgraduate Hospital Educational Environment Measure).

Although researchers agree that the larger the sample size, the better for the CFA, there is no universal agreement on sufficient size. A sample of over 200 is considered acceptable for most models [28, 30, 27]. Other authors have proposed a minimal number of cases for each question (5 per question) [36, 37]. Our sample study of 211 meets the requirements of the aforementioned rules.

There are several indices of goodness of fit, and most of them can be interpreted as describing the lack of fit of the model to the data [30]. Each type of adjustment index provides different information about the fit of the model (or the non-fit), so that researchers generally indicate several indices of fit when evaluating the fit of the model. There are many guidelines for an “acceptable” model fit [28, 38]. For this study, we used the threshold values recommended by Brown. It is important to note that these are not rigid guidelines, and Brown comments that his use of “close to” for threshold values is intentional [29]. The most used indices of goodness of fit are SRMR, RMSEA, CFI and TLI [2, 13, 24]. The SRMR (Standardized Root Mean Square Residual) is an absolute fit index that is based on the discrepancy between the correlations in the input matrix and the correlations predicted by the model [29, 30]. RMSEA (root mean square error of approximation) is a parsimony correction indice that tests the extent to which the model fits reasonably well in the population [29, 30]. We found that the SRMR (0.058) had a good fit (< 0.06), and RMSEA (0.07) had an acceptable fit (< 0.10), indicating that the tested model fits well to the data [16, 24]. Both comparative fit indices, CFI (0.88) and TLI (0.87) were close to the 0.90 threshold, indicating a limited improvement of the tested model relative to a restricted, nested baseline model [29, 30]. Silkens et al. [13] reported better fit results with SRMR and RMSEA of 0.04 (good result), CFI and TLI at 0.92 and 0.91 respectively, which is considered acceptable. Boor et al. [2] obtained a CFI of 0.89 (near acceptable threshold) and a RMSEA of 0.04 (good) and considered this result as indicating a good fit of the model (Table 3). Only two studies attempted to validate the DRECT instrument in a non-European context [16, 24]. In the Philippines, Pacifico et al. did not obtain a good fit of the model proposed by Silkens et al. [13]. They proposed an alternative model with 28 questions that gave better model fit results. Dominguez et al. assessed the reliability, construct validity and concurrent validity of the DRECT in the Spanish language in Columbia [24]. Given our results, and compared to the results obtained by Boor and Silkens, we can consider that we obtained an adequate model fit. This study therefore validated the French version of the DRECT instrument, thus allowing its use in French-speaking countries.

One of the challenges of medical research is the reproducibility of the scientific results obtained. In this case, reproducibility of the results obtained by the original authors suggests the robustness of the DRECT instrument and its adaptability to other international residency programs.

One of the limitations of this study was the small size of our sample compared to previous studies [2, 13, 16]. Nevertheless, this is relative since the size of our sample is sufficient to carry out a confirmatory factor analysis [29]. Another limitation is the absence of a strong tool for content validity in this study such as the percentage of agreement by independent reviewers, or content validity index [27, 39].

## Conclusion

This study enabled the psychometric validation of the French translation of the Dutch Residency Educational Climate Test and may be used to evaluate the learning climate in French-speaking contexts. Further research is needed in order to confirm these results in other French-speaking countries.

## Supplementary information

**Additional file 1.** French version of the Dutch Residency Educational Climate Test.

**Additional file 2.** Statistics for each item of the DRECT (mean, standard deviation, Discrimination, factor loadings).

## Data Availability

Dataset for this study is available under reasonable request.

## Abbreviations

DRECT: The Dutch Residency Educational Climate Test
SRMR: Standardized root mean square residual
RMSEA: The root mean square error approximation
CFI: The Comparative Fit Index
TLI: Tucker- Lewis Index
DREEM: Dundee Ready Education Environment Measure
PHEEM: Postgraduate Hospital Educational Environment Measure
AVE: Average variance extracted
CFA: Confirmatory factor analysis
