# Supplementary material for: Validation of the French translation of the Dutch residency educational climate test
Source: BMC Med Educ. 2020 Oct 2;20:338. doi: 10.1186/s12909-020-02249-4 (PMC7531085; doi:10.1186/s12909-020-02249-4)
Supplement: Supplementary file 1 — Additional file 1. French version of the Dutch Residency Educational Climate Test. [file 12909_2020_2249_MOESM1_ESM.pdf]

Supplementary material 1: French translation and adaptation of the DRECT

Subscales	Items
**Atmosphère éducative**	*1. La continuité des soins des patients n'est pas affectée par les différences d'opinions entre les encadrants (enseignants)*
	2. Les différences d'opinions entre les encadrants (enseignants) au sujet de la prise en charge des patients, sont discutées de manière instructive pour les autres personnes présentes.
	3. Les différences d'opinion n'ont pas un impact négatif sur le climat de travail
	4. Aucun encadrant n'a un impact négatif sur le climat de formation.
	5. Mes encadrants me traitent avec respect.
**Le travail en équipe**	*6. Les encadrants, le personnel infirmier, les autres professionnels de la santé et les résidents travaillent ensemble en équipe.*
	7. Le personnel infirmier et les autres professionnels de la santé apportent une contribution positive à ma formation.
	8. Le personnel infirmier et les autres professionnels de la santé sont disposés à réfléchir avec moi sur la prestation des soins aux patients
**Rôle du Chef de service***	*9. Le chef de service surveille les progrès de ma formation.*
	10. Le chef de service fournit des conseils à d'autres encadrants en cas de besoin.
	11. Le chef de service est activement impliqué dans l'amélioration de la qualité de l'enseignement et de la formation.
	12. Durant ce stage, les évaluations incluent des discussions utiles sur mes performances.
	13. Mes projets pour l'avenir font partie de ces discussions (lors des évaluations).
	14. L'avis de plusieurs encadrants est pris en considération lors des évaluations
**Coaching et évaluation**	*15. Mes encadrants prennent l'initiative d'évaluer ma performance.*
	16. Mes encadrants prennent l'initiative d'évaluer les situations difficiles dans lesquelles j'ai été impliqué.
	17. Mes encadrants évaluent si ma performance dans la prise en charge des patients correspond à mon niveau de formation
	18. Mes encadrants m'observent occasionnellement quand j'interroge un patient
	19. Mes encadrants évaluent non seulement mon expertise médicale mais aussi d'autre compétences telles que le travail d'équipe, l'organisation ou le comportement professionnel
	20. Mes encadrants donnent des Feedback réguliers sur mes forces et mes faiblesses
**Enseignement et formation**	*21. Les résidents sont généralement en mesure d'assister aux activités d'enseignement programmées (exemple: cours, staffs…)*
	22. Les activités d'enseignement se déroulent comme planifié.
	23. Les encadrants contribuent activement à la présentation d'un contenu académique de grande qualité.
	24. Les activités d'enseignement et de formation académiques sont appropriées à mes besoins
**Collaboration entre résidents**	*25. Les résidents travaillent bien ensemble*
	26. Les résidents, en tant que groupe, s'assurent que le travail de la journée a été réalisé.
	27. Au sein de notre groupe de résidents, il est facile de trouver quelqu'un pour assurer ou échanger une garde
**Le travail est adapté à la compétence des résidents.**	*28. Le travail que je fais est adapté à mon niveau d'expérience.*
	29. Le travail que je fais correspond à mes objectifs d'apprentissage à ce stade de ma formation.
	30. Il est possible de suivre l'évolution des patients.

**Accessibilité des Encadrants**	*31. Lorsque j'ai besoin d'un encadrant, je peux toujours en contacter un.*
	32. Quand j'ai besoin d'avoir l'avis d'un encadrant, ils sont facilement disponibles.
	33. L'encadrant qui me supervise est clairement identifié
**La sortie du patient****	*34. La sortie des patients est utilisée comme une opportunité d'enseignement*
	35. Les encadrants encouragent les résidents à participer à la discussion lors de la sortie des patients

* The subscale "Role of speciality tutor" was translated as "Role of the department head to be adapted to Moroccan residency context.

** Patient sign-out subscale: was difficult to understand and translate for the research team. It was adapted to "Patient discharge" which is often used as a teaching opportunity in the residency context.
