# Supplementary material for: Validation of the French translation of the Dutch residency educational climate test
Source: BMC Med Educ. 2020 Oct 2;20:338. doi: 10.1186/s12909-020-02249-4 (PMC7531085; doi:10.1186/s12909-020-02249-4)
Supplement: Supplementary file 2 — Additional file 2. Statistics for each item of the DRECT (mean, standard deviation, Discrimination, factor loadings). [file 12909_2020_2249_MOESM2_ESM.pdf]

Supplementary material 2: Statistics for all DRECT items

Items	Mean	SD	Rit	Corrected Item-total correlation	Factor loadings
**Educational atmosphere** *(Atmosphère éducative)*					
Continuity of care is not affected by differences of opinion between attendings. *La continuité des soins des patients n'est pas affectée par les différences d'opinions entre les encadrants (enseignants)*	3.213	1.178	0.67	0.606	0.64
Differences of opinion between attendings about patient management are discussed in such a manner that is instructive to others present. *Les différences d'opinions entre les encadrants (enseignants) au sujet de la prise en charge des patients, sont discutées de manière instructive pour les autres personnes présentes.*	3.531	1.164	0.66	0.593	0.69
Differences of opinion are not such that they have a negative impact on the work climate *Les différences d'opinion n'ont pas un impact négatif sur le climat de travail*	2.919	1.253	0.75	0.673	0.71
There is (are) NO attending physician(s) who have a negative impact on the educational climate. *Aucun encadrant n'a un impact négatif sur le climat de formation*	2.659	1.290	0.7	0.629	0.71
My attendings treat me with respect. *Mes encadrants me traitent avec respect*	3.493	1.140	0.66	0.589	0.72
**Teamwork** *(Le travail en équipe)*					
Attendings, nursing staff, other allied health professionals and residents work together as a team. *Les encadrants, le personnel infirmier, les autres professionnels de la santé et les résidents travaillent ensemble en équipe.*	3.185	1.134	0.63	0.566	0,69
Nursing staff and other allied health professionals make a positive contribution to my training *Le personnel infirmier et les autres professionnels de la santé apportent une contribution positive à ma formation*	3.114	1.103	0.79	0.705	0,80
Nursing staff and other allied health professionals are willing to reflect with me on the delivery of patient care. *Le personnel infirmier et les autres professionnels de la santé sont disposés à réfléchir avec moi sur la prestation des soins aux patients*	2.924	1.296	0.76	0.666	0.87
**Role of specialty tutor** *(Rôle du Chef de service)*					
The specialty tutor monitors the progress of my training *Le chef de service surveille les progrès de ma formation*	3.289	1.286	0.86	0.821	0.87
The specialty tutor provides guidance to other attendings when needed. *Le chef de service fournit des conseils à d'autres encadrants en cas de besoin*	3.562	1.116	0.79	0.754	0.8
The specialty tutor is actively involved in improving the quality of education and training. *Le chef de service est activement impliqué dans l'amélioration de la qualité de l'enseignement et de la formation*	3.427	1.316	0.86	0.815	0.87
In this rotation, evaluations are useful discussions about my performance *Durant ce stage, les évaluations incluent des discussions utiles sur mes performances.*	3.085	1.232	0.85	0.81	0.84
My plans for the future are part of the discussion. *Mes projets pour l'avenir font partie de ces discussions (lors des évaluations)*	2.682	1.203	0.76	0.726	0.76
During evaluations, input from several attendings is considered. *L'avis de plusieurs encadrants est pris en considération lors des évaluations*	3.199	1.158	0.68	0.65	0.68
**Coaching and assessment** *(Coaching et évaluation)*					
My attendings take the initiative to evaluate my performance. *Mes encadrants prennent l'initiative d'évaluer ma performance*	3.18	1.178	0.82	0.781	0.84
My attendings take the initiative to evaluate difficult situations I have been involved in. *Mes encadrants prennent l'initiative d'évaluer les situations difficiles dans lesquelles j'ai été impliqué.*	3.047	1.202	0.87	0.813	0.87
My attendings evaluate whether my performance in patient care is commensurate with my level of training *Mes encadrants évaluent si ma performance dans la prise en charge des patients correspond à mon niveau de formation*	3.18	1.209	0.84	0.789	0.84
My attendings occasionally observe me taking a history. *Mes encadrants m'observent occasionnellement quand j'interroge un patient*	2.716	1.248	0.69	0.659	0.67
My attendings assess not only my medical expertise but also other skills such as teamwork, organization or professional behavior. *Mes encadrants évaluent non seulement mon expertise médicale mais aussi d'autre compétences telles que le travail d'équipe, l'organisation ou le comportement professionnel*	3.071	1.327	0.81	0.773	0.8

My attendings give regular feedback on my strengths and weaknesses<br>*Mes encadrants donnent des Feedback réguliers sur mes forces et mes faiblesses*	2.847	1.322	0.83	0.794	0.82
**Formal education** *(Enseignement et formation)*					
Residents are generally able to attend scheduled educational activities.<br>*Les résidents sont généralement en mesure d'assister aux activités d'enseignement programmées (exemple: cours, staffs…)*	3.531	1.122	0.74	0.699	0,72
Educational activities take place as scheduled.<br>*Les activités d'enseignement se déroulent comme planifié.*	2.877	1.205	0.78	0.736	0.77
Attendings contribute actively to the delivery of highquality formal education.<br>*Les encadrants contribuent activement à la présentation d'un contenu académique de grande qualité*	3.052	1.266	0.87	0.813	0.89
Formal education and training activities are appropriate to my needs.<br>*Les activités d'enseignement et de formation académiques sont appropriées à mes besoins*	2.938	1.219	0.85	0.794	0.89
**Resident peer collaboration** *(Collaboration entre résidents)*					
Residents work well together.<br>*Les résidents travaillent bien ensemble*	3.531	1.070	0.82	0.737	0,87
Residents, as a group, make sure the day's work gets done.<br>*Les résidents, en tant que groupe, s'assurent que le travail de la journée a été réalisé.*	3.526	1.101	0.76	0.681	0,80
Within our group of residents, it is easy to find someone to cover or exchange a call.<br>*Au sein de notre groupe de résidents, il est facile de trouver quelqu'un pour assurer ou échanger une garde*	3.573	1.004	0.65	0.591	0.66
**Work is adapted to residents' competence** *(Le travail est adapté à la compétence des résidents)*					
The work I am doing is commensurate with my level of experience.<br>*Le travail que je fais est adapté à mon niveau d'expérience.*	2.924	1.127	0.72	0.589	0,77
The work I am doing suits my learning objectives at this stage of my training.<br>*Le travail que je fais correspond à mes objectifs d'apprentissage à ce stade de ma formation.*	2.957	1.232	0.78	0.649	0,83
It is possible to do follow up with patients<br>*Il est possible de suivre l'évolution des patients.*	3.678	1.042	0.41	0.355	0,48
**Accessibility of supervisors** *(Accessibilité des Encadrants)*					
When I need an attending, I can always contact one.<br>*Lorsque j'ai besoin d'un encadrant, je peux toujours en contacter un.*	3.943	0.969	0.88	0.768	0,87
When I need to consult an attending, they are readily available.<br>*Quand j'ai besoin d'avoir l'avis d'un encadrant, ils sont facilement disponibles.*	3.787	1.031	0.87	0.753	0,92
It is clear which attending supervises me.<br>*L'encadrant qui me supervise est clairement identifié*	3.668	1.278	0.61	0.582	0,63
**Patient sign-out** *(La sortie du patient)*					
Sign-out is used as a teaching opportunity.<br>*La sortie des patients est utilisée comme une opportunité d'enseignement*	2.957	1.176	0.84	0.791	0,85
Attendings encourage residents to join in the discussion during sign-out.<br>*Les encadrants encouragent les résidents à participer à la discussion lors de la sortie des patients*	3.123	1.244	0.84	0.791	0.93
SD: standard deviation					
Rit: Discrimination or Item-total correlation					
